# Supplementary material for: Genetic Diagnosis for 64 Patients with Inherited Retinal Disease
Source: Genes (Basel). 2022 Dec 26;14(1):74. doi: 10.3390/genes14010074 (PMC9859429; doi:10.3390/genes14010074)
Supplement: Supplementary file 1 [file genes-14-00074-s001.zip › genes-2070474-supplementary.pdf]

Supplemental Table S1. Patient demographics

ID	Gender	Ethnicity	Age at Dx/ GT	Dx	Inheritance	Refraction OD; OS	BCVA OD; OS	LogMAR OD; OS
DM001	M	WC	3/56	CRD	Dominant	ND	20/320; CF	1.2;
DM002	M	WC	5/8	RP	Recessive	+2.50+1.00x090; +1.75+2.00x090	20/50; 20/63	0.4; 0.5
DM003	F	WC	13/16	MD	Recessive	-5.50+1.00x090; -5.00+1.25x090	20/100; 20/100	0.7; 0.7
DM004	M	WC	23/28/	CRD	Recessive	ND	20/400; 20/125	1.3; 0.8
DM005	M	NA	35/50	MD	Dominant	-1.75+1.75x040; -1.50+0.75x2.25	20/25; 20/32	0.1; 0.2
GP001	F	WC	33/37	MD	Isolate	ND	20/20; 20/20	0; 0
GP002	F	BA,NA	49/51	RP	Recessive	ND	20/50; 20/40	0.4; 0.3
GP004	F	WC	35/36	MD	Dominant	-0.25+4.50x100; +1.25+1.00x077	20/32; 20/20	0.2; 0
GP005	M	WC	25/46	MD	Recessive	ND	20/50; 20/160	0.4; 0.9
GP005a	F	WC	10/50	MD	Recessive	ND	20/800; 20/640	1.6; 1.5
GP006	M	WC,NA	15/21	RP	X-Linked	-3.75+1.75x100; -3.75+1.00x096	20/160; 20/125	0.9; 0.8
GP006a	F	WC,NA	10/41	RP	X-Linked	+0.25-1.25x045; -2.75-3.75x155	20/32; 20/500	0.2; 1.4
GP006b	F	NA	35/64	RP	X-Linked	0.00+3.50x105; -3.00+4.75x080	20/32; 20/30	0.2; 0.2
GP008	F	WC	25/51	RP	Recessive	ND	20/50; 20/50	0.4; 0.4
GP008a	F	WC	30/55	RP	Recessive	ND	20/50; 20/50	0.4; 0.4
GP009	M	WC	39/53	RP	Dominant	+0.75+0.50x170; +1.25+0.25x165	20/320; 20/200	1.2; 1
GP012	M	Asian	31/42	CRD	Dominant	ND	20/250; 20/640	1.1; 1.5
GP013	F	WC	35/36	MD	Dominant	ND	20/20; 20/20	0; 0
GP013a	F	WC,BA	20/21	MD	Dominant	-0.50+0.25x075; Plano	20/15; 20/20	-0.12; 0
GP014	M	WC	60/71	CRD	Recessive	-8.25+1.00x130; -9.75+1.25x055	20/640; 20/200	1.5; 1
GP015	F	WC	40/79	RP	Isolate	ND	20/100; 20/200	0.7; 1
GP016	M	WC	7/25	RP	X-Linked	+0.50+1.00x080; : 0.00+1.00x095	20/63; 20/80	0.5; 0.6
GP017	M	NA	20/48	MD	Isolate	+3.00-1.00x124; +3.50-0.75x076	20/200; 20/200	1; 1

GP018	M	NA	20/56	RP	Isolate	ND	20/200; 20/400	1; 1.3
GP019	F	UK	69/70	MD	Dominant	+1.00+0.00x000; Plano	20/320; 20/32	1.2; 0.2
GP020	M	WC	22/23	RP	Dominant	-0.25+0.75x110; -0.25+0.50x050	20/25; 20/25	0.1; 0.1
GP021	M	NA	5/68	CRD	Recessive	-7.50+2.75x080; -7.25+2.50x120	20/400; 20/320	1.3; 1.2
GP022	M	NA	48/49	RP	Dominant	0.00+0.75x135; +0.25+0.00x000	20/25; 20/63	0.1; 0.5
GP022a	F	NA	27/36	RP	Dominant	-6.00+0.50x090; -6.25+1.00x105	20/20; 20/25	0; 0.1
GP026	F	WC	41/41	MD	Recessive	-1.75+0.75x120; -3.00+1.25x085	20/125; 20/160	0.8; 0.9
GP028	F	WC	25/84	RP	Isolate	ND	HM; 20/125	; 0.8
GP029	M	WC	80/81	RP	X-Linked	ND	LP; LP	
GP031	F	WC	30/70	MD	Recessive	-2.75+00x000; -3.00+0.00x000	20/60; 20/160	0.48; 0.9
GP032	M	WC	37/51	RP	Recessive	+0.25+0.75x010; -0.75+0.75x145	20/20; 20/20	0; 0
GP034	M	WC	35/59	RP	Recessive	0.00+0.75X025; NLP	20/25; NLP	0.1;
GP035	F	NA	39/41	MD	Dominant	-5.25+0.00x000; -5.75+2.00x090	20/40; 20/25	0.3; 0.1
GP036	F	NA	20/70	RP	Dominant	-2.25+1.50x126; -3.25+0.75x014	CF; CF	
GP037	F	WC	30/55	RP	Isolate	+1.00+1.00x146; +0.50+1.25x012	20/63; 20/63	0.5; 0.5
GP039	F	WC	27/40	RP	Isolate	-0.25+0.75x110; 0.00+0.75x070	20/32; 20/32	0.2; 0.2
GP044	M	WC	40/65	RP	Dominant	-2.50 +0.50x085; -1.00 +1.00x082	20/50; 20/80	0.4; 0.6
GP045	F	WC	34/53	RP	Recessive	ND	20/20; 20/20	0; 0
GP045a	F	WC	36/42	RP	Recessive	-1.50+1.50x065; -1.25+1.00x090	20/40; 20/40	0.3; 0.3
GP046	F	WC	23/24	MD	Isolate	+4.00+0.75x015; +1.25+0.75x117	20/32; 20/63	0.2; 0.5
GP047	F	WC	60/67	MD	Recessive	-4.00-2.25x010; -2.50-2.25x170	20/40; 20/40	0.3; 0.3
GP047a	M	WC	14/48	MD	Recessive	-2.50 +1.75x112; -2.00 +1.50x065	20/40; 20/50	0.3; 0.4
GP048	M	BA	31/49	MD	Isolate	-2.00+0.00x000; -2.50+0.50x150	20/20; 20/20	0; 0
GP049	M	NA	40/49	RP	Recessive	-4.25+0.00x000; -4.25+0.50x175	20/50; 20/32	0.4; 0.2
GP050	M	WC	47/49	MD	Recessive	+2.75+0.75x150; +2.50+1.00x011	20/32; 20/25	0.2; 0.1
GP052	F	WC	13/26	MD	Recessive	-2.50+1.00x090; -2.50+1.25x090	20/160; 20/160	0.9; 0.9

GP053	M	WC	31/34	MD	Recessive	-1.75+0.75x090; -2.25+1.00x090	20/15; 20/15	-0.1; -0.1
GP054	F	WC	29/32	RP	Dominant	-1.00+0.00x000; -2.00+0.00x000	20/25; 20/25	0.1; 0.1
GP058	F	WC	24/70	RP	Dominant	-0.25+1.75x180; -0.50+1.00x020	20/125; 20/40	0.8; 0.3
GP059	F	BA	46/47	MD	Isolate	-0.50+0.50x020; -0.75+0.50x165	20/20; 20/20	0; 0
GP060	M	WC	53/67	MD	Recessive	-0.50+0.50x020; 0.00+0.50x150	20/80; 20/63	0.6; 0.5
GP061	F	WC	21/28	RP	Isolate	ND	20/200; 20/160	1; 0.9
GP062	F	WC	33/44	MD	Isolate	unknown	20/20; 20/20	0; 0
GP063	M	WC	16/35	RP	X-Linked	+0.50+1.25x120; 0.00+1.25x060	20/320; 20/200	1.2; 1
GP063a	F	WC	30/60	RP	X-Linked	+0.75+0.00x000; Plano	20/20; 20/40	0; 0.3
GP064	F	WC	35/39	MD	Isolate	+3.25+0.00x000; +3.00+1.00x160	20/20; 20/20	0; 0
GP065	M	WC	51/57	CRD	Isolate	-1.75+1.50x155; -2.00+1.50x180	20/100; 20/125	0.7; 0.8
GP066	F	BA	31/46	RP	Recessive	-3.25+0.75x175; -4.75+0.75x025	20/40; 20/15	0.3; -0.1
GP067	F	WC	50/62	CRD	Dominant	-1.75+0.50x174; -3.00+0.50x090	20/200; 20/200	1; 1
GP067a	M	WC	38/35	CRD	Dominant	-5.50+1.50x100; -4.00+1.00x171	20/40; 20/25	0.3; 0.1

M, Male; F, Female; Dx, Diagnosis; GT, Genetic Testing; WC, White/Caucasion; BA, Black/African American; NA, Native American; UK, Unknown/undisclosed; MD, Macular Dystrophy; RP, Retinitis Pigmentosa; CRD, Cone-Rod Dystrophy; ND, not done; OD oculus dexter (right eye); OS oculus sinister (left eye); CF, counting fingers; HM, hand motion; LP, light perception

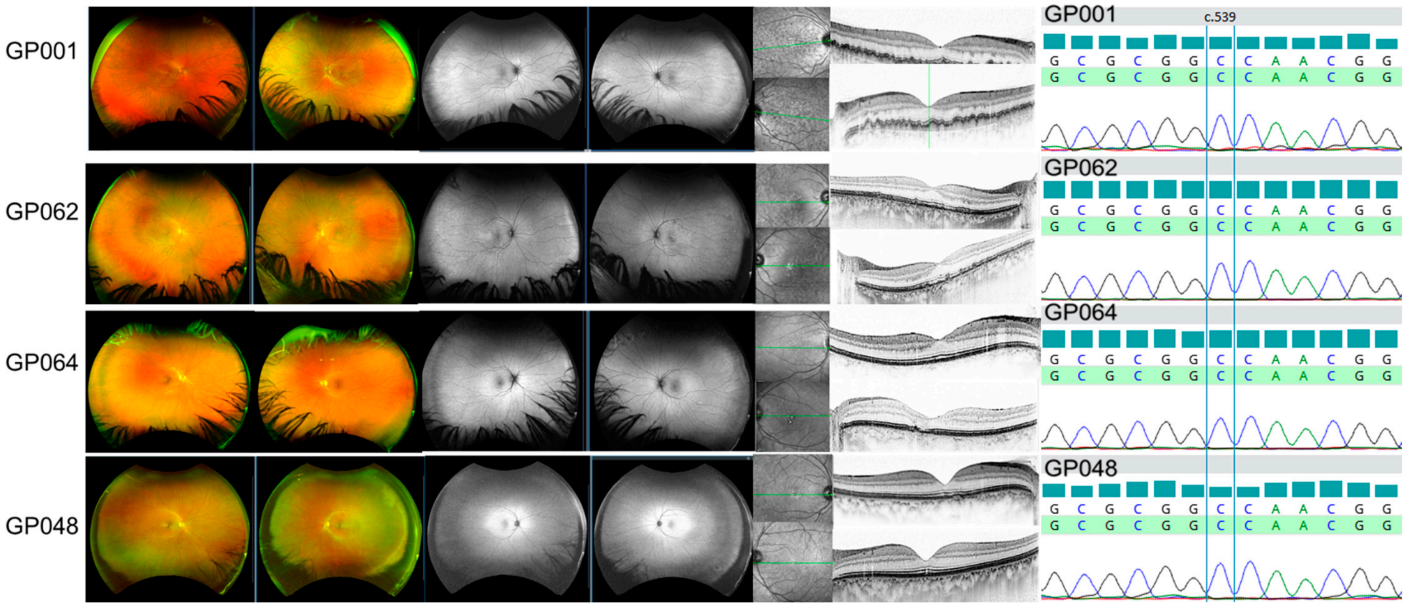

Supplemental Figure S1: The c.539 location in *CLEC3B* associated with macular disorder is wild type in four patients with similar clinical features. Drusen can be seen in the posterior pole with rod involvement determined by nyctalopia and some decreased rod response of ffERG[34]. Sanger sequencing was performed for these four unrelated individuals following PCR amplification of their genomic DNA.
